# Circular RNAs in leukemia

**DOI:** 10.18632/aging.102091

**Published:** 2019-07-15

**Authors:** Zijuan Wu, Handong Sun, Jianyong Li, Hui Jin

**Affiliations:** 1 Department of Hematology, The First Affiliated Hospital of Nanjing Medical University, Jiangsu Province Hospital, Nanjing 210029, China; 2 Key Laboratory of Hematology of Nanjing Medical University, Nanjing 210029, China; 3 Collaborative Innovation Center for Cancer Personalized Medicine, Nanjing 210029, China; 4 The First Affiliated Hospital of Nanjing Medical University, Nanjing 210029, China

**Keywords:** non-coding RNAs, circRNAs, leukemia, aging disease, biomarker, therapy

## Abstract

In pace with the development of gene sequencing technology and transcriptome research, it has been found that 70 to 90% of the human genome is transcribed into RNAs, while only 2% of RNAs encode proteins. This implies that non-coding RNAs (ncRNAs) may exert vital biological functions and a full analysis of non-coding transcriptomes is needed. Over the past decade, the advance in high-throughput sequencing and transcriptome profiling has enabled the identification of circular RNAs (circRNAs) involved in many biological processes and the occurrence and development of diseases. Accumulating evidence has revealed that circRNAs may serve as new biomarkers for diagnosis as well as provide promising therapeutic approaches and novel drug screening strategies for leukemia. A comprehensive understanding of circRNAs in leukemia is a prerequisite for the development of clinical translational research. In this review, we will discuss the general information of circRNAs and focus on the current advances in understanding the association between dysregulated circRNAs and leukemia.

## circRNAs BACKGROUND

**Figure 1:**
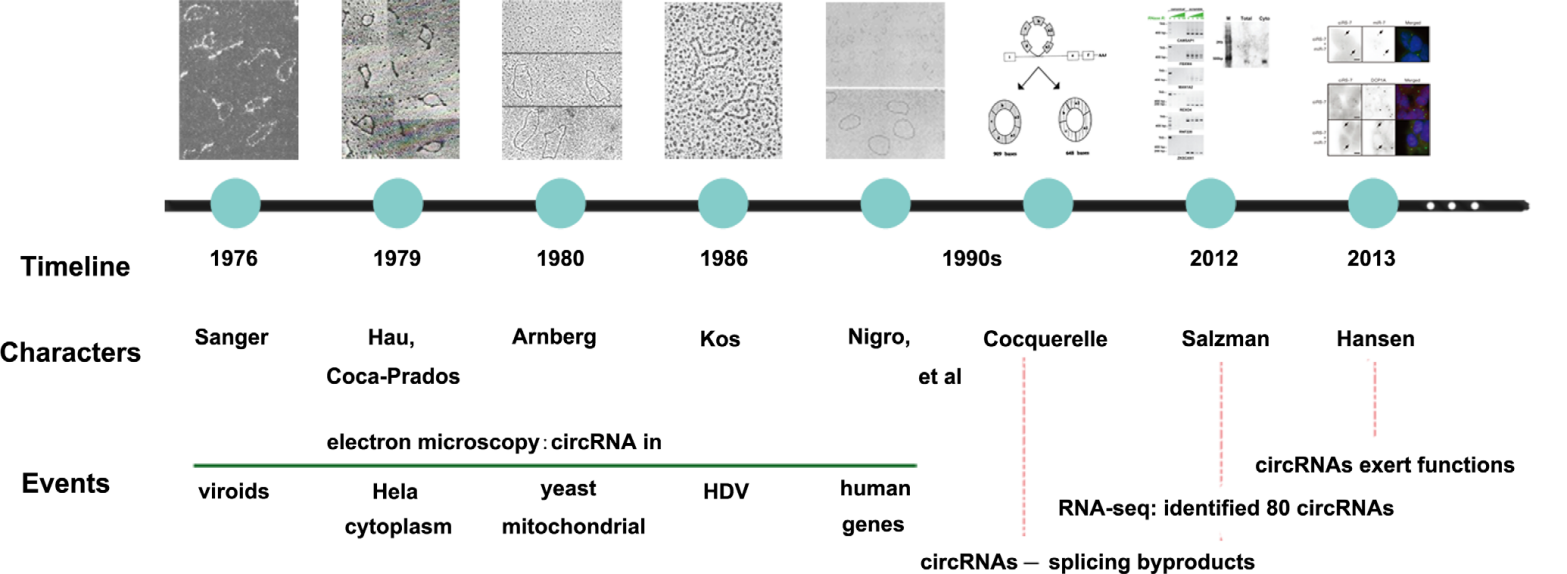
**The development history of circRNAs.** The figure represents a summary of important events leading to the discovery of circRNAs.

In 1976, since Sanger [1] discovered a closed circular single-stranded RNA molecule with covalent bonds in viroids through electron microscopy, the concept of circRNAs came into being for the first time. Three years later, Hsu and Coca-Prados [2] extracted RNA from Hela cytoplasm, and the electron microscopy revealed that 1-2% of them were circRNAs. They also suggested that the majority of circRNAs may localize in cytoplasm rather than in nucleus. In 1980, Arnberg [3] observed that about 50% of the yeast mitochondrial RNA which was separated by agarose gel electrophoresis from 11S and 18S fragments contained circular molecules and was also confirmed in saccharomyces cerevisiae by ten years [4]. In 1986, Kos [5] presented HDV contained a single-stranded circular RNA molecule. As early as 1990s, first endogenous circRNA with inverted order of exons was found in human DCC by Nigro [6], followed by human proto-oncogene ests-1 and mice Sry genes [7,8]. Before 2000s, circRNAs were regarded as either splicing byproducts or intermediates escaping lariat structures from debranching [8–10]. Not until 2012, along with the mushroom growth of high-throughput sequencing and microarray technology as well as the application of bioinformatics, circRNAs had begun to be recognized as large species of RNAs with thousands of members in mammalian cells. Salzman [11] first reported about 80 circRNAs based on RNA sequencing results. Subsequently, more than 25,000 circRNAs in human fibroblasts, 1,950 circRNAs in HEK293 cells, 1,903 circRNAs in mouse brain tissue and 724 circRNAs in nematodes were found [12]. Thus, circRNAs are thought to be important transcriptional products. Danan has found abundant circRNAs which might have certain biological functions [13]. Memczak [14] presented the opinion that coding sequences possessed previously unrecognized regulatory potential. In addition, Hansen’ s [15] study, the first functional analysis of a naturally expressed circRNA, together with above-mentioned study, has aroused researchers’ intense interest in circRNAs research. The main development trends of circRNAs were shown in Figure 1.

CircRNAs, a family of single-strand ncRNAs with covalently closed circular isoforms, are generated from precursor mRNAs (pre-mRNAs) [16] and can be regulated by specific RNA splicing factor which are involved in ‘backsplicing’ reaction [17]. Other than functioning as miRNA sponges [18–20], circRNAs could exert their bioactivities by interacting with proteins [21,22]. For example, circ-Foxo3 was found to be associated with cell cycle progression and proliferation via forming ternary complexes with p21 and CDK2 [23]. In addition, circRNAs may play essential roles in regulating transcription and alternative splicing [24,25]. CircRNAs that mainly distribute in cytoplasm promote transcription by interacting with the Pol II complex [26].What’s more, circRNAs can guide protein translation [27]. Abe [28] reported that circRNAs in cell-free Escherichia coli translation system could be efficiently translated by a rolling circle amplification (RCA) mechanism. Legnini [29] reported circ-znf609 could directly encode proteins and participate in the process of muscle development. More biological functions and significance of circRNAs are still under exploring.

**Figure 2:**
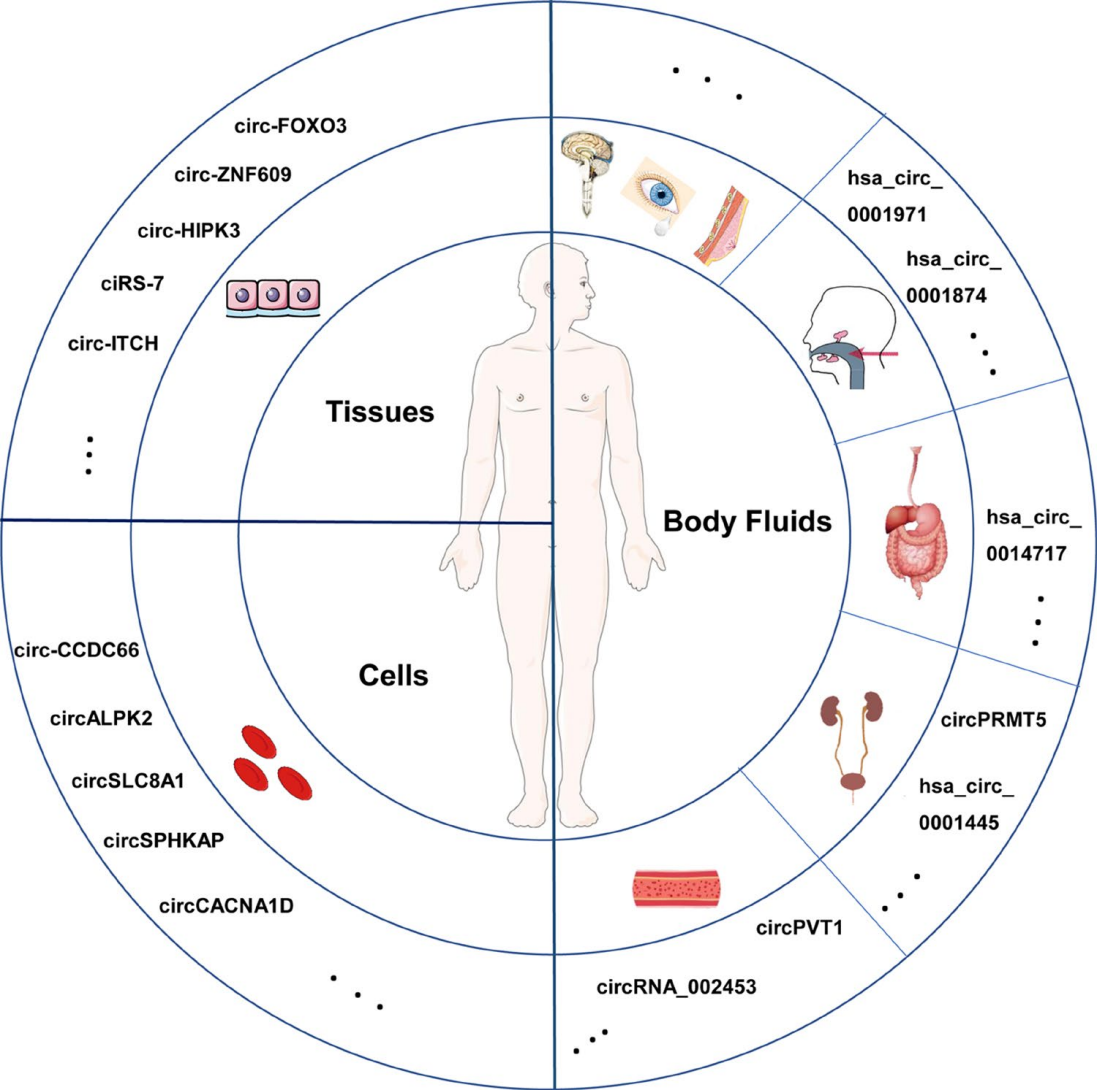
**Different sources of circRNAs in human.** CircRNAs are widespread in multiple human specimens, such as tissues, cells and diverse body fluids. Several circRNAs are listed as examples and dots in the circles represent circRNAs that have been identified and that still need to be explored.

Based on present study, widespread presence of circRNAs derived from various types of human samples is elucidated (Figure 2). It lays the foundation for unlimited potential of circRNAs. Xu [30] conducted a systematic examination of circRNAs in various human normal tissues. Further analysis suggested that circRNAs that closely related to biological functions were widely distributed and their expression was developmental stage- and tissue-specific. For instance, the 332 circRNAs enriched in bowel, colon and large intestine probably functioned in the digestive system, while the 243 circRNAs abundant in prostate and thyroid might play critical roles in male reproducing or development [31]. Some circRNAs with abnormal expression in cancer tissues were revealed to be involved in tumorigenesis, progress and metastasis and serve as possible biomarkers and new targets for treatment [32,33]. There is evidence that plentiful circRNAs are components of blood cells [11]. Bonizzato [34] reported numbers of circRNAs in blood cells at different stages of hematopoietic differentiation, corroborating earlier suggestions that circRNAs expression were developmental stage- and tissue-specific. Moreover, abundantly presented in tumor cells, circRNAs are verified to play roles in tumor cell proliferation and metastasis [35]. CircRNAs are also detected in body fluid such as blood samples [36], cerebrospinal fluid [37], saliva [38], gastric juice [39] and even urine [40]. As a surrogate of non-invasive biomarker, circRNAs may have a chance to shine in the future. In addition, circRNAs are believed to present in human tears, milk and other body fluids. It will be quite an exciting news if circRNAs in body fluids can be verified individual-specific or disease-specific, which will alleviate patients’ pain to a large extent in clinical tests, providing helpful information for diagnosis and treatment.

## INTRODUCTION TO LEUKEMIA

**Figure 3:**
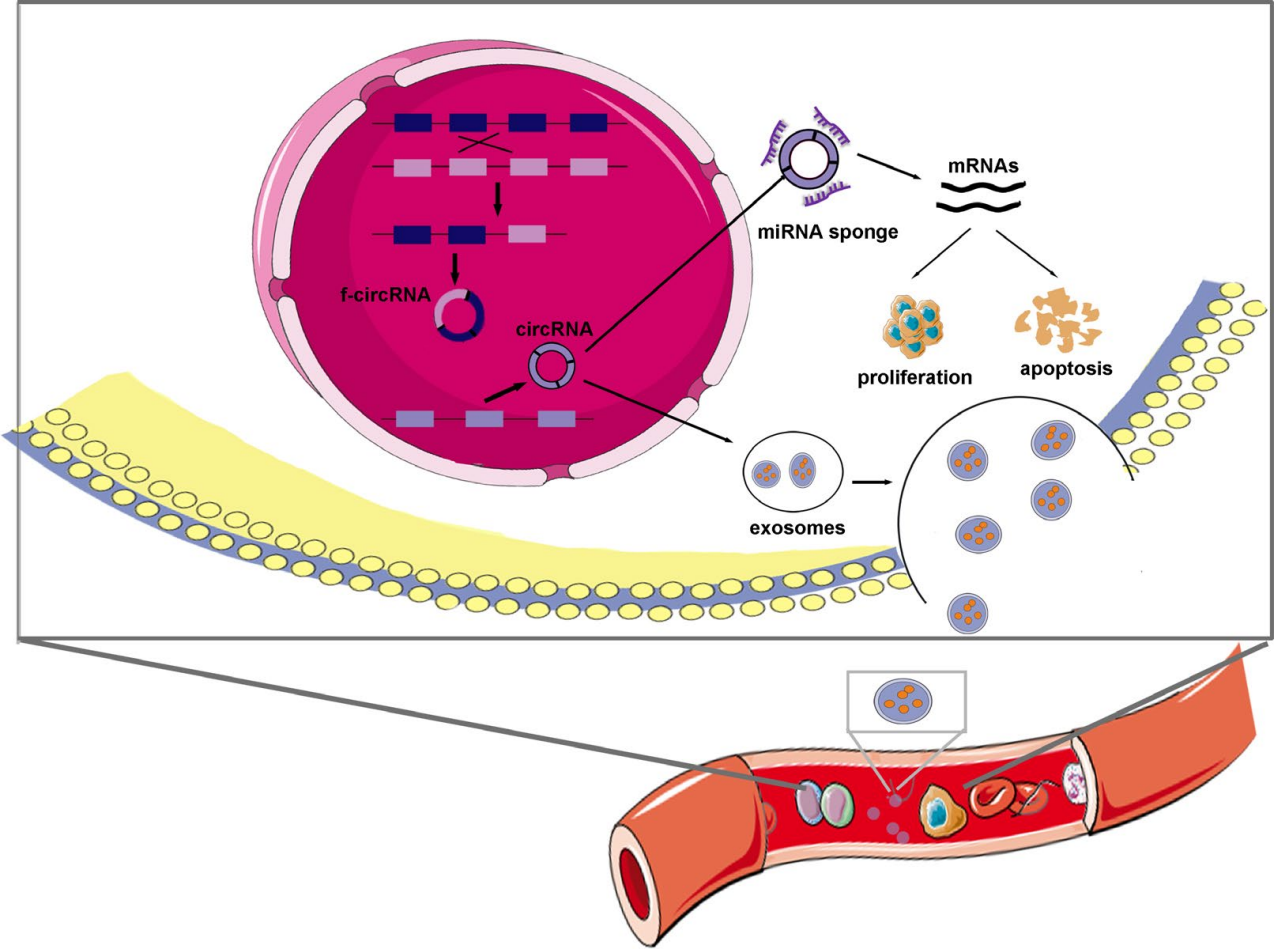
**Schematic representation of the proposed mechanism of circRNAs in leukemia.** CircRNAs and f-circRNAs could be transcribed separately from genes and fusion genes. CircRNAs that predominantly distribute in cytoplasm, play parts in leukemia mainly via sponging specific miRNAs and thus modulating mRNAs roles in cell proliferation and apoptosis. Exosomes are possible transport vectors that facilitate circRNAs circulating in blood and others and subsequently promoting leukemogenesis and progression.

Leukemia is a life-threatening cancer with cloned hematopoietic stem cells and most often diagnosed in adults (mid 60s). The most common types of leukemia are acute myeloid leukemia (AML), acute lymphoblastic leukemia (ALL), chronic myeloid leukemia (CML) and chronic lymphocytic leukemia (CLL). With no clear preventive or control measures at present, leukemia is still a malignant disease and a threat to public health. Several studies have noted the important role of ncRNAs, such as microRNAs (miRNAs) and long non-coding RNAs (lncRNAs) in the pathogenesis of leukemia [41–43]. CircRNAs are expressed in both normal and malignant hematopoietic cells. In a certain sense, circRNAs have important implications for induced pluripotent stem cell (iPSC) modeling in hematopoietic system diseases [34]. It appears that circRNAs have more diverse expression profiles in cancer cells and may influence the protein expression levels of hematopoietic cell genes at different stages. Although overwhelming evidence has illustrated that circRNAs are of great importance to leukemia biogenesis, maintenance, and progression, a specific overview on circRNAs in leukemia is lacking. To comprehensively understand the functions and regulatory mechanisms of circRNAs in leukemia, we reviewed the remarkable and ground-breaking leukemia studies (Figure 3, Table 1).

**Table 1:** CircRNAs in leukemia.

**Leukemia types**	**circRNA** **(circBase ID)**	**Host gene**	**Expression**	**Source**	**miRNA sponge (target gene/pathway)**	**Functions**	**Clinical significance**	**Reference**
AML	f-circPR, f-circM9_1	PML/RARα, MLL/AF9	upregulated	BM	-	Facilitate cell transformation, vitality and resistance to treatment	Potential diagnostic and therapeutic implications	[44]
AML	circ-PVT1 (hsa_circ_0001821)	PVT1	upregulated	PB	let-7 or miR-125 families*	Involved in the development of leukemia	Help to explore biological mechanisms behind MYC amplifications	[46]
AML	circNPM1 75001 (hsa_circ_0075001)	NPM1	upregulated	BM	miR-181 family/ TLR signaling pathway*	Associated with myeloid differentiation	A potential biomarker for classification and risk stratification	[51]
AML	circ-HIPK2 (hsa_circ_0001756)	HIPK2	downregulated	PB	miR-124-3p	Regulate ATRA-induced differentiation	A potential biomarker	[52]
AML	circ-ANAPC7 (has_circ_101141)	ANAPC7	upregulated	BM	miR-181 family*	Participate in the pathogenesis of AML	A promising diagnostic biomarker and novel drug target	[54]
AML	circRNA-DLEU2 (hsa_circ_0000488)	DLEU2	upregulated	BM	miR-496/PRKACB	Promote cell proliferation and inhibited cell apoptosis	A novel biomarker and therapeutic target	[55]
AML	hsa_circ_100290	SLC30A7.	upregulated	BM	miR-203/Rab10	Promote cell proliferation and inhibited cell apoptosis	A potential diagnostic and therapeutic target	[56]
AML	hsa_circ_0004277	WDR37	downregulated	BM	miR-138-5p, miR-30c-1-3p, miR-892b, miR-571, miR-328-3p/SH3GL2, PPARGC1A, PIP4K2C, SH2B3, ZNF275, and ATP1B4*	Associated with risk-status and treatment	A potential diagnostic biomarker	[62]
AML	circ_0009910 (hsa_circ_100053)	MFN2	upregulated	BM	miR-20a-5p.	Promote cell proliferation, cell cycle arrest and inhibit c629ell apoptosis	A novel outcome biomarker and potential therapeutic targets	[63]
AML	circ-PAN3 (hsa_circ_0100181)	PAN3	upregulated	BM	miR-153-5p/miR-183-5p/XIAP	Mediate the development of drug resistance	A valuable indicator for predicting clinical efficacy and potential target for reversing drug resistance	[64].
ALL	circPVT1	PVT1	upregulated	BM	let-7, miR-125*/ Bcl-2, c-Myc	Promote cell proliferation inhibit cell apoptosis	A potential therapeutic target	[70]
CML	circBA9.3	BCR-ABL1	upregulated	PB	-	Promote cell proliferation, TKI resistance and inhibit apoptosis	A potential diagnostic and therapeutic target for TKI-resistant patients	[73]
CLL	circ-CBFB (hsa_circ_0000707)	CBFB	upregulated	CLL cells	miR-607/*FZD3*/Wnt/β-catenin	Promote CLL cell proliferation, cell cycle progression and inhibit cellular apoptosis	An effective diagnostic and prognostic biomarker	[77]
CLL	circ_0132266 (hsa_circ_0132266)	MTO1	downregulated	CLL cells	miR-337-3p/PML	Inhibit CLL cell proliferation, and promote apoptosis	An effective diagnostic and prognostic biomarker	[78]

This table summarizes circRNAs that have been identified in human leukemia (AML, ALL, CML, CLL) and their roles in diagnosis, prognosis, disease progression, drug response evaluation. Information including host genes, expression levels, miRNA targets (direct and indirect), clinical values are shown (*Not validated experimentally; -Not mentioned).

### Acute Myeloid Leukemia (AML)

AML is the most common acute leukemia in adults. The incidence of AML increases with age and patients show significantly multifaceted biological and clinical heterogeneity. Recent studies have revealed the value of circRNAs in AML. While the expression pattern of circRNAs might be impaired anomalous in leukemia cells, altered circRNAs could be involved in leukemogenesis as well [44].

The cytogenetic and molecular genetic characteristics are significant prognostic factors for AML patients. In some cases, circRNAs make us better understand the pathogenic mechanism of AML. Despite the fact that some genomic amplicons are related to poor prognosis, the internal structures of amplicon formation and their potential mechanisms in AML have not yet been clarified yet. 8q24 amplifications are associated with aberrant chimeric genes and could generate oncogenic lncRNAs that play functional roles in leukemia [45]. Circ-PVT1, generated from exon 2 of PVT1 on chromosome 8, is often accompanied by 8q24 amplicons [46]. Circ-PVT1 was reported to act as a molecular sponge for tumor suppressor miRNAs, such as let-7 family [47] or miR-125 family [48]. The expression level of circ-PVT1 in AML patients with amplifications (AML-amp) was significantly higher than the patients with normal karyotype. The discovery of posttranscriptional chimeras and circRNAs involved with 8q24 amplified genes in AML-amp cases could broaden our exploration of the underlying mechanisms of in human leukemia.

Nucleophosmin (*NPM1*) was regarded as one of the most frequently mutated genes in AML. Bearing both proto-oncogene and tumor-suppressing properties, *NPM1* could be involved in ribosomal biogenesis, apoptosis, and cell proliferation by encoding a multifunctional chaperone protein. The mutation of *NPM1* might contribute to AML [49,50]. Given the relevance of *NPM1* in AML and aiming to deepen the understanding of multifaceted gene, Hirsch [51] explored the circRNA variants from *NPM1* through RNA-Seq-based transcriptome analysis. In *NPM1* wild-type and mutated AML patients, circNPM1 75001 was highly expressed in AML patients. Although circNPM1 75001 was independent of the *NPM1* mutational status, a positive correlation was found between the expression of circNPM1 and total *NPM1*. Li’s study also suggested an independent relationship between circRNAs and their linear host mRNAs [52]. Overall, these findings would contribute to distinguishing different AML subgroups based on circRNA expression profile and offering new insight into exploring the biological characteristics of leukemia cells.

Fusion genes formed by two unrelated genes are very common in cancer genomes. Displaying adverse effects, fusion genes are important cancer markers in diseases [53]. In leukemia, chromosomal translocation often leads to gene fusion. The discovery of fusion circRNAs (f-circRNAs) and the study of their functions has created a new insight into understanding the mechanisms of leukemia and tailoring therapies for patients. Guarnerio [44] studied two types of leukemia, acute promyelocytic leukemia (APL) with translocation between PML and RARα, and AML with translocation between MLL and AF9. Based on the concept that one single gene can generate more than one circRNA, they elucidated that the corresponding fusion genes could produce f-circRNAs. Moreover, f-circRNAs can facilitate cell transformation, vitality and resistance to treatment. While f-circRNAs alone were not enough to trigger leukemia, they appeared to work with other cancer-promoting signals, such as MLL-AF9 fusion protein, to promote disease progression. It suggested that circRNAs, such as protein genes, are also affected by the rearrangement of genes, resulting in abnormal fusions. These aberrant f-circRNAs further contribute to the development of leukemia and may be an important target for antitumor drugs. However, the specific molecular mechanism of the function of f-circRNAs requires intensive study.

CircRNAs can serve as novel promising diagnosis biomarkers for AML. For example, circ-ANAPC7 which was validated upregulated frequently in AML patients was proposed to be a potential indicator for AML diagnosis [54]. circRNA-DLEU2 [55] and hsa_circ_100290 [56] were also potential biomarkers identified and verified to be highly expressed in AML patients. Consistent with the commonly reported competitive endogenous RNA (ceRNA) mechanism, circRNA-DLEU2 promoted cell proliferation and reproduction through the miR-496/PRKACB axis. Hsa_circ_100290 reported to function in oral squamous cell carcinomas [57] was revealed to act as an oncogene in AML occurrence and progression, promoting cell proliferation and inhibiting apoptosis at the same time. To elucidate the potential mechanism of hsa_circ_100290, researchers further screened for its targeted miRNAs. As expected, hsa_circ_100290 can affect cell functions via regulating miR-203/Rab10 axis. Approximately 45–50% of AML cases showed no obvious genomic alterations when assessed using conventional banding analysis [49,58]. To diagnose and distinguish APL patients from cytogenetically normal (CN) AML patients, circRNA profiles were analyzed using acfs (accurate circRNA finder suite). A set of differentially expressed circRNAs were identified, and the results illustrated that circRNAs could facilitate the molecular stratification of AML [59]. The multiple research has identified novel genetic alterations and provided promising biomarkers for AML diagnosis and classification.

Aside from being biomarkers in leukemia, circRNAs could also instruct the treatment and predict prognosis of patients. Extramedullary infiltration (EMI), which always corresponds with relapse and refractory, is relatively common in AML [60]. Given that EMI is closely correlated with poor prognosis in AML patients, Lv [61] carried out circRNA microarrays in bone marrow mononuclear cells from EMI and non-EMI AML patients to uncover novel abnormal moleculars. 17 circRNAs were noted to act as key regulators of cell–cell crosstalk in EMI, and most of their target genes could predicted poor prognosis. Several molecular abnormalities were considered as crucial factors affecting AML treatment effect. To offer a potential diagnostic marker and treatment target in AML, Li [62] performed an analysis using a microarray platform and intensity filtering system. Through a comparative analysis of AML patients and healthy patients, hsa_circ_0004277 was selected from characterized circRNA transcripts. It was found to be aberrantly decreased in CN-AML patients but significantly elevated after treatment. It is suggested that hsa_circ_0004277 was strongly associated with the treatment of AML. In addition, circ_0009910 was found to highly express in AML. And higher expression levels of circ_0009910 usually predicted worse prognosis [63]. One of the obstacles in AML patients survival is the resistance to chemotherapeutic drugs. A high-throughput circRNA microarray was conducted to compare the expression profiles of circRNAs in chemo-sensitive and resistant AML cells. As a result, 49 circRNAs were noted to be significantly differentially expressed. Additionally, circPAN3 was confirmed to have a vital function in driving drug resistance through circPAN3-miR-153-5p/miR-183-5p-XIAP axis [64].

**Figure 4:**
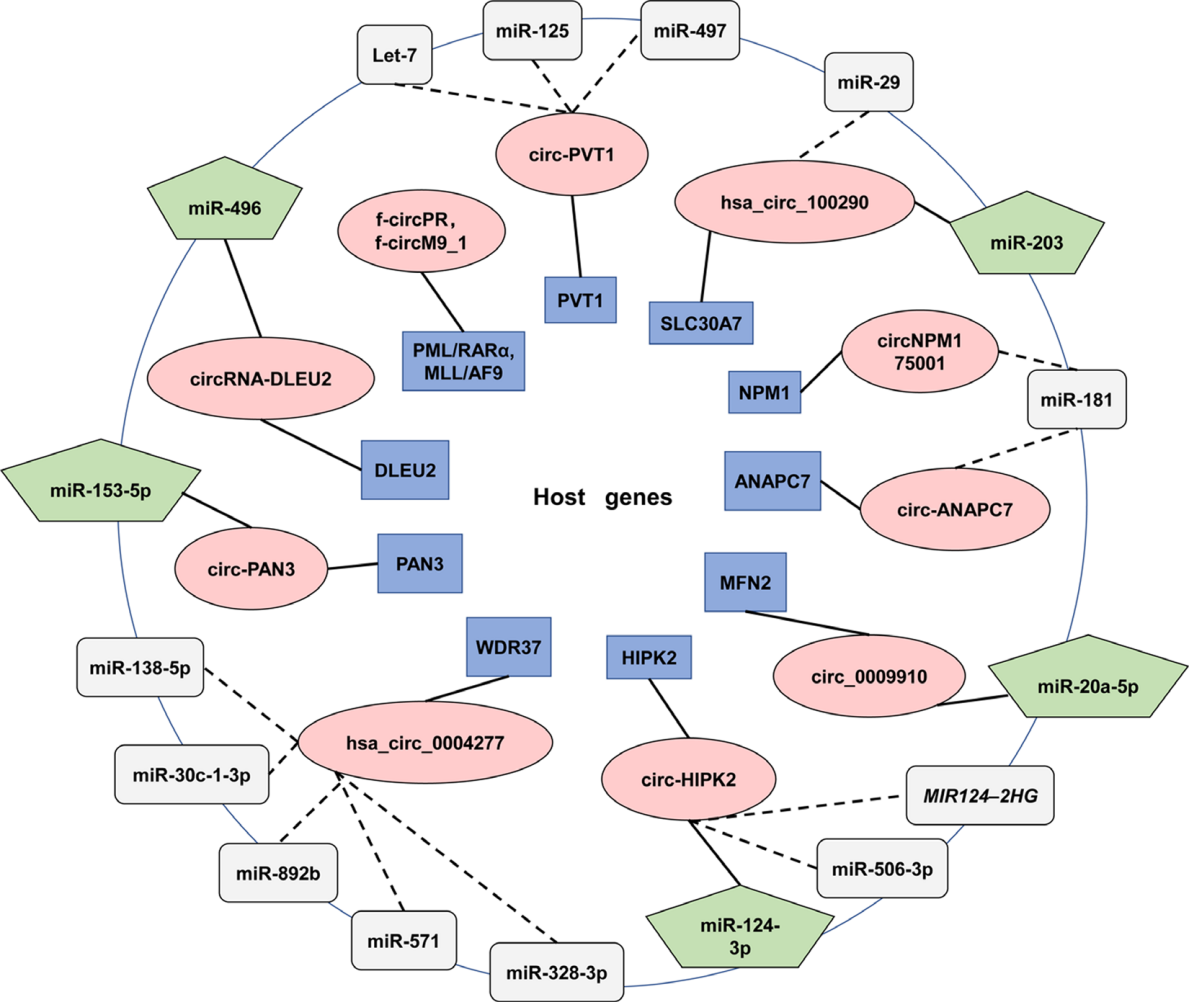
**CircRNAs and their correlation with miRNAs in AML.** The figure summarizes the reported circRNAs that functioning in AML. The innermost circle described host genes (Blue rectangle) and the red ovals in the middle are circRNAs that transcribed from them. The outermost circle exhibited targeted miRNAs. Solid lines connect circRNAs and their target miRNAs (Green pentagon). Dashed lines are used to connect miRNAs (Grey box) that have not been validated experimentally or proposed to interact with circRNAs in other diseases.

The majority of circRNAs were reported to alleviate the effects induced by miRNAs. Regulatory relationships between circRNAs, miRNA and their target genes were deciphered in Figure 4. To sum up, the expression levels of circRNAs are conductive to clinical diagnosis, treatment and prognosis assessment.

### Acute Lymphoblastic Leukemia (ALL)

ALL is a lymphoid malignancy affecting the B or T lineages. Its incidence peaks are between ages 2 and 5 years and the elders [65]. Emerging evidence shows that circRNAs may confer in ALL and have potential values in indicating diagnosis and therapy.

Based on RNA-seq results in hyperdiploid B-cell precursor ALL samples, circRNAs were found to be produced from one specific gene and differentially expressed in cells and diseases [66]. For example, circPVT1 which was reported to be involved in gastric cancer [67], AML [46] and other diseases [48,68,69] was also specifically highly expressed in ALL. Silencing of circPVT1 induced cell cycle arrest and apoptosis of ALL cells via targeting its neighbor gene c-Myc and increasing the expression level of anti-apoptotic protein Bcl-2 [70]. In addition, it has been reported that circRNAs generated from genes that related to ALL and B-cell differentiation (*JAK2, PAX5, IKZF1, ETV6 and EBF1*) are prevalently present in hyperdiploid leukemia compared with normal leukocytes samples [34].

Adults and senile patients of AML are often companied with poor outcomes. Although most children with ALL can be cured, a small number of patients who relapse after treatment have poor prognosis [66]. Given the fact that the circRNA expression pattern was dysregulated in ALL patients, efforts should be made to better understand its molecular mechanisms in ALL occurrence and relapse.

### Chronic Myeloid Leukemia (CML)

Chronic myeloid leukemia (CML) is a clonal myeloproliferative neoplasm originating from abnormal pluripotent stem cells. The oncogene *BCR-ABL1* which encodes a hyperactive tyrosine kinase is the major molecular factor for the pathogenesis of CML [71]. Imatinib, a specific tyrosine kinase inhibitor (TKI), has brought hope for the majority of CML patients. A small proportion of patients, however, have developed resistance to imatinib [72]. Recently, circRNAs have been implicated in the progression of CML and development of TKI resistance and opened up new opportunities for CML therapy.

To explore the internal mechanism of chemo-resistance and remove the obstacles to cure patients, f-circRNA circBA9.3 derived from the *BCR-ABL1* fusion genes was analyzed. Of note, circBA9.3 which increased the protein expression levels of ABL1 and BCR-ABL1 was associated with TKI resistance. Over-expressed in leukemia cells, circBA9.3 could facilitate cell proliferation and drug resistance. It is inspiring that circBA9.3 may serve as a promising diagnostic biomarker and therapeutic target for CML patients [73].

### Chronic Lymphocytic Leukemia (CLL)

CLL is a heterogeneous disease characterized by the monoclonal expansion of B cells. It is the most common adult leukemia in the West and the median age at diagnosis is 71 years [74,75]. Because of its insidious onset and significant heterogeneity, the molecular details of CLL are still under investigation.

Based on the RNA-seq data, 52 circRNAs were identified and the role of one circRNA in distinguishing different B-cell malignancies was suggested [76]. Hsa_circ_0000707, termed circ-CBFB, derived from *CBFB* on chromosome 16 and aberrantly over-expressed in CLL patients, was determined to promote cell proliferation and suppress apoptosis through miR-607/FZD3/Wnt/β-catenin pathway [77]. Given the connection between the signaling cascade and CLL progression, circ-CBFB may become a potential therapeutic target for CLL treatment. Specific miRNA in different diseases may exhibit different expression levels. To figure out why miR-337-3p was downregulated in CLL, Wu [78] has elucidated possible regulatory mechanism that circ_0132266 participated in the downregulating of miR-337-3p expression, which opens a new avenue for exploring the interaction between specific miRNA and circRNAs underlying CLL. Taken together, these data has demonstrated that circRNAs may serve as oncogenes or tumor suppressors and participate in CLL progression.

Although the molecular mechanisms of circRNAs are poorly understood in leukemia, they are indeed implicated in cellular processes and leukemia progression. Encouragingly, the exploration of aberrant circRNAs has provided us with valuable insight into the pathogenesis of hematological malignancies.

## Perspectives

Originated from human cancer xenografts, circRNAs could enter the circulation by exosomes and be easily detected in serum or plasma. Exosomes are small extracellular vesicles (EVs) (30–150 nm in size) [79] that are produced by normal or cancerous cells, including leukemia blasts [80,81]. They may play pleiotropic roles in regulating signaling transduction and supporting the growth and survival of leukemia cells by delivering mRNAs, ncRNAs and proteins between neighboring or distant cells [82,83]. Exosomes can provide not only diagnostic and prognostic information for leukemia but also antileukemic treatment modalities [84,85]. Released vesicles, such as exosomes and microvesicles, might be possible vehicles by which circRNAs are expulsed to extracellular space because enriched circRNAs have been detected in EVs [86,87]. Researchers have also discovered hundreds of circRNAs in cellular that can be transferred to exosomes and proposed that exosomal circRNAs (exo-circRNAs) traffic through a complex mechanism [88]. Furthermore, since EVs can be taken up by other cells, excreted circRNAs could mediate intercellular communication [89]. Given that exosomes can carry circRNAs and transfer them out of cells while retaining the integrity of their structures, we consider that circRNAs who are abundantly and stably present in the blood circulatory system may play nonnegligible roles in leukemogenesis, progression and drug resistance. However, little is known about exo-circRNAs in leukemia. Herein, much efforts are needed to comprehensively understand the roles of circRNAs.

A big step has been taken toward understanding the pathogenesis of leukemia since f-circRNAs have been identified and verified to promote leukemogenesis and other diseases [44,90]. Chromosomal translocations are responsible for the onset of many types of cancers, such as leukemia. These discoveries strongly arouse our interest in seeking more f-circRNAs that have great impacts on disease development, even independent of linear transcripts and fusion proteins. Notably, many studies have shown the potential of circRNAs as promising therapeutic targets. Encouragingly, an artificial circRNA that serves as miRNA “sponge” has been designed in vitro to treat and cure Hepatitis C. It can not only adsorb miRNA but also adhere to binding proteins, fulfilling their role in preventing viral infection [91]. Once artificial circRNA is presented as a medical treatment, it will highly possible to find novel and better treatments for leukemia and many other diseases.

## Conclusions

In this review, we summarize the latest research progress of circRNAs and highlight their biological significance and clinical values in leukemia. CircRNAs have been shown to play nonnegligible roles as gene regulators and perform crucial biological functions via ceRNA mechanism in leukemia. Other than indicating the occurrence of leukemia, circRNAs have the potential to be indicators of disease stage and progression and could be developed as medication for clinical use. With more in-depth study, we expect that circRNAs can be more appropriately and precisely used in clinical diagnosis and treatment in the future.
